# Biomolecular Condensates in Disease: Decoding the Material State and Engineering Precision Modulators

**DOI:** 10.3390/ijms27020837

**Published:** 2026-01-14

**Authors:** Biwei Han, Boxian Li, Xingyue Wang, Liang Wang

**Affiliations:** Key Laboratory of Pesticide & Chemical Biology of Ministry of Education, School of Life Sciences, Central China Normal University, Wuhan 430079, China; biwhan@mails.ccnu.edu.cn (B.H.); liboxian@mails.ccnu.edu.cn (B.L.); xxgz@mails.ccnu.edu.cn (X.W.)

**Keywords:** biomolecular condensates, phase separation, material state, reverse engineering, molecular grammar, precision medicine

## Abstract

The recognition of liquid–liquid phase separation (LLPS) as a widespread organizing principle has revolutionized our view of cellular biochemistry. By forming biomolecular condensates, cells spatially orchestrate reactions without membranes. However, the dysregulation of this precise physical organization is emerging as a driver of diverse pathologies, collectively termed “Condensatopathies.” Unlike traditional proteinopathies defined by static aggregates, these disorders span a dynamic spectrum of material state dysfunctions, from the failure to assemble essential compartments to the formation of aberrant, toxic phases. While research has largely focused on neurodegeneration and cancer, the impact of condensate dysfunction likely extends across broad physiological landscapes. A central unresolved challenge lies in deciphering the “molecular grammar” that governs the transition from functional fluids to pathological solids and, critically, visualizing these transitions in situ. This “material science” perspective presents a profound conundrum for drug discovery: how to target the collective physical state of a protein ensemble rather than a fixed active site. This review navigates the evolving therapeutic horizon, examining the limitations of current pharmacological approaches in addressing the complex “condensatome.” Moving beyond inhibition, we propose that the future of intervention lies in “reverse-engineering” the biophysical codes of phase separation. We discuss how deciphering these principles enables the creation of programmable molecular tools—such as synthetic peptides and state-specific degraders—designed to precisely modulate or dismantle pathological condensates, paving the way for a new era of precision medicine governed by soft matter physics.

## 1. Introduction: Towards a ‘Material Science’ Perspective of Cellular Pathology

A persistent question in cell biology concerns the mechanisms that impose order on the crowded and complex intracellular milieu, ensuring the precise spatiotemporal control of biochemical reactions [1]. While membrane-delimited organelles represent one well-established solution, cells also employ a diverse array of non-membranous bodies—from the prominent nucleolus to transient stress granules—to achieve compartmentalization [2]. Historically, the understanding of these structures emerged from the convergence of two distinct research trajectories. The first, rooted in the study of the Nuclear Pore Complex (NPC), resolved the paradox of rapid yet selective transport by proposing a “selective phase” model. Pioneering biophysical studies demonstrated that disordered phenylalanine-glycine (FG) repeat domains of nucleoporins form a cohesive hydrogel barrier that functions as a selective molecular sieve, excluding inert macromolecules while permitting the passage of transport receptors [3,4,5,6,7]. The second trajectory, originating from the study of RNA-binding proteins (RBPs) in germ granules, revealed that low-complexity domains (LCDs) can assemble into “reversible fibers” to drive the formation of dynamic liquid droplets [8,9,10,11].

These paths have unified into the current paradigm: biomolecular condensates formed via the physical process of liquid–liquid phase separation (LLPS) [9,12] represent a ubiquitous organizing principle [13,14]. This “material science” perspective revealed that multivalent macromolecules, frequently utilizing low-complexity domains (LCDs) or broader intrinsically disordered regions (IDRs), can undergo spontaneous demixing from the bulk cytoplasm or nucleoplasm, analogous to the separation of oil and water [15,16]. Far from being a specialized mechanism, LLPS appears to be a ubiquitous strategy across life, playing roles in organizing processes as diverse as gene expression, DNA repair, intracellular signaling, and immune responses [17,18].

The formation, dissolution, material characteristics (such as viscosity and viscoelasticity), and biological roles of these condensates are subject to intricate regulation, governed by what can be termed a “molecular grammar” encoded within macromolecular sequences and modulated by the surrounding cellular environment [19]. Disruptions to this finely tuned homeostasis, whether arising from genetic lesions, environmental insults, or the systemic decline associated with aging, can precipitate disease. This growing realization has spurred the adoption of the unifying term “Condensatopathies” to describe disorders originating from the dysregulation of biomolecular condensates [20].

It is crucial to distinguish this concept from the more established notion of “proteinopathy” (e.g., amyloidosis). While proteinopathies typically focus on the pathological consequences of stable, often fibrillar, protein aggregates representing an end-stage structure, condensatopathy describes a disease affecting the *entire lifecycle and dynamic behavior* of these phase-separated assemblies [21]. Pathogenesis in condensatopathies can manifest through various deviations from normal function. These include a Loss-of-Function (LOF) scenario, where essential condensates fail to assemble correctly, thereby impairing downstream processes. Pathology can also arise from detrimental alterations in the material properties of condensates—for instance, a shift from a fluid, dynamic state to a more viscous, gel-like state that hinders molecular exchange and function, even preceding the formation of irreversible solid structures. Furthermore, Toxic Gain-of-Function (TGOF) mechanisms occur when aberrant condensates form, potentially sequestering vital cellular components, acting as ectopic signaling hubs, or directly exerting cytotoxic effects.

This conceptual reframing—viewing cellular organization and disease through the lens of material science—opens up exciting new avenues but also presents formidable challenges. How does the cell precisely tune the ‘material state’ of its internal compartments? What biophysical principles govern the transition between functional fluidity and pathological rigidity? And, critically, how can we therapeutically intervene in processes governed by complex physical chemistry rather than discrete enzymatic active sites? Despite the explosion of research in this area, translating these fundamental insights into effective clinical strategies has proven difficult, suggesting fundamental obstacles remain. This review aims to delineate these key challenges, examining the gaps in our knowledge of the disease landscape, the mechanistic intricacies of pathological transitions, and the limitations of current therapeutic approaches, while proposing directions for future innovation.

## 2. Charting the Landscape: The Unexplored Breadth of Condensate Pathology

Our initial explorations into the realm of condensatopathies have been largely guided by prominent examples, particularly the roles of proteins like TDP-43, FUS, and Tau in devastating neurodegenerative diseases such as Amyotrophic Lateral Sclerosis (ALS), Frontotemporal Dementia (FTD), and Alzheimer’s Disease (AD). This focus, while yielding invaluable knowledge, has inadvertently created a “lamp-post” effect, potentially obscuring the true extent of diseases driven by condensate dysfunction. Accumulating evidence strongly suggests that these well-characterized cases represent only the visible portion of a much larger, mostly submerged, pathological “iceberg.” A comprehensive summary of the expanding landscape of condensatopathies across diverse physiological systems is provided in Table 1.

The potential vastness of this landscape was strikingly highlighted by a comprehensive bioinformatic analysis, which linked over 36,000 pathogenic variants across more than 1200 Mendelian disorders and numerous cancers to the *plausible* disruption of biomolecular condensate formation or function [22]. This finding serves as a compelling indicator that a significant number of human ailments may possess an underlying, yet currently unrecognized, etiology rooted in faulty phase separation. Indeed, compelling examples are emerging far beyond the confines of the nervous system.

### 2.1. Oncogenic Condensates: Hijacking and Sequestration

Cancer biology provides a particularly rich area where condensate dysregulation is emerging as a central theme. Here, pathogenesis frequently involves a TGOF mechanism, not typically leading to solid aggregation, but rather the formation of “oncogenic condensates.” These aberrant structures function as neomorphic hubs that hijack and fundamentally rewire cellular programs, particularly transcription and signaling. Oncogenic fusion proteins generated by chromosomal translocations often exemplify this principle. In acute myeloid leukemia (AML), the NUP98-HOXA9 fusion utilizes the phase separation-prone domains of NUP98 to form ectopic nuclear bodies. These bodies act as potent recruitment platforms, concentrating transcriptional co-activators like p300 and the Mediator complex at target gene loci, thereby establishing hyper-activated transcriptional states that fuel leukemogenesis [22]. A similar mechanism operates in synovial sarcoma, where the SS18-SSX fusion forms aberrant condensates on chromatin, displacing normal regulatory complexes and hijacking the BAF chromatin remodeling complex to drive pathological gene expression programs [23]. Beyond hijacking, aberrant condensates can also promote cancer by sequestering and inactivating tumor suppressors. For instance, studies have shown that nuclear condensates formed by the splicing factor SFPQ can physically entrap the tumor suppressor Smad4, thereby insulating it from upstream signals and effectively silencing the anti-proliferative TGF-β pathway [24]. These examples underscore a critical point: targeting the *assembly* or *function* of these oncogenic condensates may offer therapeutic avenues for cancers driven by proteins lacking conventional druggable pockets.

### 2.2. Developmental Disorders: The Consequence of Failed Assembly

**Figure 1:**
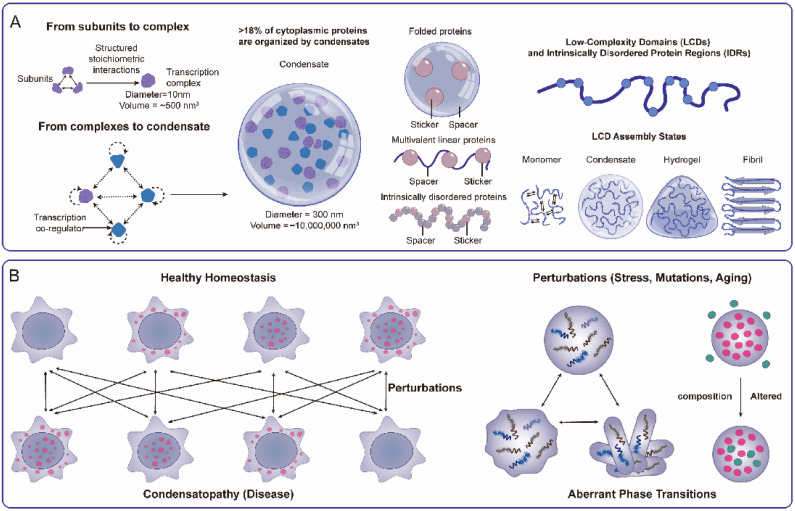
The Biophysical Basis and Classification of Condensatopathies. (**A**) The Molecular Grammar of Condensates. (**Left**) A comparison of biological organization scales, contrasting the classical paradigm of stable, stoichiometric macromolecular complexes (1–10 nm) with the emerging paradigm of mesoscale biomolecular condensates (100–1000 nm) driven by dynamic, multivalent interactions. (**Middle**) A schematic of the “Stickers-and-Spacers” molecular grammar. “Stickers” (interaction motifs within IDRs or folded domains) drive the multivalent connectivity required for phase separation, while “spacers” (flexible linkers) dictate the material properties and porosity of the resulting network. Specificity is encoded by the spatial arrangement and chemical nature of these stickers. (**Right**) The material spectrum of Low-Complexity Domain (LCD) assembly. LCDs exhibit material plasticity, existing in an equilibrium that spans disordered monomers, dynamic liquid condensates, viscoelastic hydrogels, and ordered fibrils. This ability to adopt distinct material states allows LCDs to drive diverse biological functions. (**B**) A Logic Framework for Disease Classification. A flowchart delineating the biophysical etiology of condensatopathies. The trajectory traces from upstream “Triggers” (e.g., genetic mutations, environmental stress, aging) to specific biophysical alterations (e.g., aberrant phase transitions like liquid-to-solid or compositional shifts). These defects manifest as two core pathogenic mechanisms: Loss-of-Function (LOF), defined by failed assembly or functional deficiency; and Toxic Gain-of-Function (TGOF), defined by aberrant aggregation or sequestration.

While neurodegeneration often involves the Toxic Gain-of-Function (TGOF) of aggregates, a significant category of condensatopathies arises from a primary Loss-of-Function (LOF) mechanism, particularly in neurodevelopmental disorders (Figure 1B). Development requires the exquisitely timed formation and dissolution of condensates. Rett Syndrome (RTT) serves as a canonical example. It results from mutations in MeCP2, a chromatin organizer. Recent studies demonstrate that MeCP2 normally utilizes LLPS to compact chromatin into heterochromatin condensates. RTT-causing mutations, particularly in the Methyl-Binding Domain (MBD), directly compromise this phase separation ability, leading to a failure in chromatin compartmentalization and gene silencing [25]. Similarly, Spinal Muscular Atrophy (SMA) is driven by the loss of functional SMN condensates. The SMN complex is essential for assembling spliceosomes within nuclear Cajal bodies; SMA-linked mutations disrupt this critical phase separation, leading to fatal defects in RNA processing [26].

This landscape is broadening. Emerging evidence links Neurofibromatosis type 2 (NF2) to condensate dysregulation, where mutant NF2 proteins fail to phase separate with Hippo pathway components [27]. Furthermore, progressive hearing loss has been linked to defects in the phase separation of Cdh23, which is required for tip-link stability in the inner ear [28]. Additionally, the precise balance of phase separation in synaptic proteins (e.g., FMRP, SYNGAP1) is vital for neuronal maturation, with dysregulation linked to autism spectrum disorders [17,29,30,31]. This LOF principle is recapitulated in diverse conditions, including certain ciliopathies, forms of progressive hearing loss, and myasthenic syndromes, where mutations compromise the LLPS-driven assembly of essential cellular structures [32,33].

### 2.3. Systemic Pathology: Cardiovascular, Metabolic, and Aging Implications

The influence of condensatopathies also extends significantly to cardiovascular and metabolic diseases. Cardiac function relies on the precise organization and dynamics of numerous protein assemblies. Mutations in the RNA-binding protein RBM20, which alter its phase separation behavior, lead to aberrant splicing of crucial sarcomeric proteins like titin, resulting in severe dilated cardiomyopathy [34]. Similarly, the protein HIP-55 forms condensates whose dynamics, regulated by phosphorylation, are essential for maintaining cardiac muscle fiber integrity; dysregulation of HIP-55 phase separation is directly implicated in the pathogenesis of heart failure [35].

Metabolic disorders also intersect with condensate biology. In type 2 diabetes, the aggregation of Islet Amyloid Polypeptide (IAPP), a process often nucleated via LLPS, contributes to pancreatic β-cell dysfunction. Even age-related conditions like cataracts are now being viewed through the lens of condensatopathy, resulting from the failure of chaperone systems (like α-crystallins, which themselves rely on dynamic assembly) leading to the pathological phase separation and light-scattering aggregation of lens proteins [36]. The sheer diversity of these examples—spanning oncology, cardiology, metabolic disease, and developmental biology—strongly argues that our current understanding, heavily biased towards neurodegeneration, captures only a fraction of the true clinical impact of condensate dysregulation. A major imperative for the field is thus to develop and deploy systematic, unbiased discovery platforms capable of mapping the full “condensatome” of human disease, moving beyond the current “lamp-post” limitations and revealing the vast, unexplored territory of potential therapeutic targets. A comprehensive summary of the expanding landscape of condensatopathies across diverse physiological systems is provided in Table 1.

**Table 1:** The Expanding Landscape of Condensatopathies: From Neurodegeneration to Systemic Disorders.

Disease Category	Protein/Driver	Pathogenic Mechanism (LOF/TGOF)	Biophysical Defect & Consequence	Key Refs.
Neurodevelopmental	MeCP2	LOF	Mutations in MBD disrupt chromatin condensation; failure to form heterochromatin leads to gene regulation defects (Rett Syndrome).	[15,25]
	SMN1	LOF	Disrupted phase separation prevents SMN complex assembly in Cajal bodies; impairs snRNP biogenesis (Spinal Muscular Atrophy).	[26]
	FMRP/SYNGAP1	LOF	Defective synaptic condensates impair mRNA transport or postsynaptic density organization; linked to Autism Spectrum Disorders.	[17,30]
	Cdh23	LOF	Failure to form stable tip-link condensates in the inner ear leads to progressive hearing loss.	[28]
Neurodegenerative	TDP-43	Double-Hit	Cytoplasmic solidification (TGOF) causes nuclear depletion (LOF); drives splicing failure (e.g., STMN2) and toxicity in ALS/FTD.	[37,38]
	FUS	TGOF	“Liquid-to-Solid” transition (LST) accelerates fibrillization; disrupts RNP granule dynamics and DNA repair.	[39,40]
	TAF15	TGOF	Forms specific amyloid filaments in Frontotemporal Lobar Degeneration (FTLD-TAF15), distinct from FUS.	[41]
Cancer	NUP98-HOXA9	TGOF (Hijacking)	Forms “oncogenic condensates” that concentrate super-enhancer machinery (p300) to drive leukemogenesis (AML).	[42]
	SFPQ	TGOF (Sequestration)	Aberrant nuclear condensates sequester tumor suppressor Smad4, inhibiting TGF-β signaling.	[24]
Cardio-Metabolic	RBM20	LOF/TGOF	Altered granular dynamics lead to mis-splicing of Titin, causing Dilated Cardiomyopathy.	[34]
	HIP-55	LOF	Impaired phosphorylation-regulated assembly disrupts cardiac muscle fiber maintenance (Heart Failure).	[35]

## 3. Deciphering the Rules: The Molecular Grammar of Homeostasis and Its Pathological Disruption

Even for the condensate-forming proteins implicated in disease, a deeper challenge remains: elucidating the precise, causal chain linking molecular alterations to pathological outcomes. Observing that a mutation correlates with aberrant condensation is only the first step. The critical question is *how* and *why* this occurs at a molecular and structural level. Understanding this requires delving into the “molecular grammar” that dictates condensate behavior and confronting the current gaps in our knowledge—the mechanistic “black box.”

### 3.1. The Framework: Homeostasis from a Tunable “Molecular Grammar”

The behavior of functional biomolecular condensates is not random; it is governed by a complex but potentially decipherable “molecular grammar” encoded primarily within the sequences of their constituent macromolecules [19] (Figure 1A). At the level of physical chemistry, the “Stickers-and-Spacers” model provides a useful conceptual framework [43]. In this view, specific “sticker” elements (such as aromatic residues mediating π-π interactions, charged motifs enabling electrostatic contacts, or structured domains involved in specific binding events) provide the attractive forces driving association. Interspersed “spacer” regions, often intrinsically disordered and flexible, modulate the valency, spatial arrangement, and conformational freedom of the stickers, thereby influencing the overall phase behavior (e.g., the critical concentration for separation) and the material properties (e.g., viscosity, surface tension) of the resulting condensate. The careful balance between sticker strength and spacer characteristics determines the condensate’s identity and function (Figure 1A), as demonstrated by detailed studies of proteins like FUS [19]. Figure 1A illustrates this hierarchical organization, contrasting the classical stoichiometric complexes with the dynamic, multivalent interactions that define condensates.

However, a purely interaction-centric view may be incomplete. Evidence suggests that many functional condensates possess an underlying, albeit dynamic, structural organization. The “reversible fiber” model, originating from studies of RNA-binding proteins with low-complexity domains, proposes that many such proteins function by assembling into labile, polymeric structures often exhibiting cross-β characteristics [8]. The key insight here is the concept of a “virtue of weakness” [10,44] and what can be termed the “Temperate Zone” hypothesis. Unlike the highly stable, irreversible β-sheets found in pathogenic amyloids, these functional polymers are proposed to employ specific structural motifs—such as “polar zippers” relying on networks of weak hydrogen bonds rather than hydrophobic packing [45], or “kinked β-sheets” that introduce geometric frustration [46]—precisely to *limit* their stability and ensure rapid reversibility. This inherent structural lability, poised at the thermodynamic edge of equilibrium, allows the condensate to readily assemble when needed and disassemble when the signal dissipates. This represents a sophisticated evolutionary solution, balancing the need for compartmentalization with the requirement for dynamic responsiveness.

### 3.2. The Pathology: When the Grammar Breaks Down

Condensatopathies arise when this finely tuned molecular grammar is disrupted, leading to assemblies that are either too weak, too strong, or incorrectly formed. Pathologies stemming from a grammar that is “too weak” typically result in LOF due to a failure of assembly. As highlighted previously in developmental disorders like Rett syndrome (impaired MeCP2 chromatin condensation) or SMA (defective SMN complex assembly), mutations can directly compromise the “sticker” interactions or alter spacer properties sufficiently to prevent the formation of a functional condensate [25,26]. Similarly, the inability to form LLPS-driven DNA repair foci due to mutations in components like FUS represents a critical LOF leading to genome instability [39] (Figure 1B).

**Figure 2:**
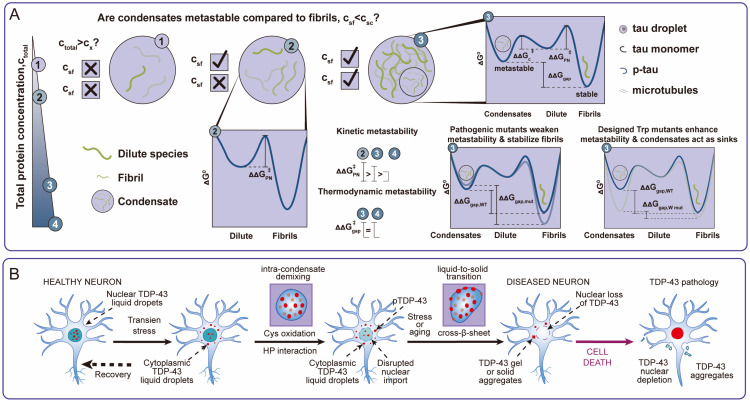
Mechanisms of Pathological Progression in Condensatopathies. (**A**) Pathological Maturation via Tunable Metastability. The “Tunable Metastability” model explains the thermodynamics of neurodegeneration and TGOF. Functional liquid condensates occupy a metastable energy state (shallow well) but can transition into thermodynamically more stable, yet pathological, amyloid fibrils (deep well). This process, often involving intra-condensate demixing or Liquid-to-Solid Transition (LST), is accelerated by disease-linked mutations in proteins such as Tau or TDP-43. Note: While rigidity is often pathological here, functional rigidity exists in specific contexts (e.g., centrosomes). (**B**) The “Double-Hit” Pathogenic Paradigm. Exemplified by TDP-43 proteinopathy, this model illustrates the vicious cycle where cytoplasmic aggregation acts as a toxic sink (TGOF), simultaneously sequestering protein away from the nucleus. This leads to the depletion of nuclear TDP-43 and a catastrophic Loss-of-Function (LOF) in essential splicing events, compounding cellular dysfunction.

More complex are the pathologies arising from a grammar that is “too strong” or “incorrect,” leading to TGOF. This often manifests as pathological maturation, particularly in neurodegenerative diseases, frequently involving a liquid-to-solid transition (LST). The concept of “tunable metastability” provides a powerful thermodynamic framework for understanding this process [47]. Functional, liquid-like condensates are viewed as occupying a shallow, *metastable* energy state—kinetically accessible and suitable for dynamic function, but not the lowest possible energy state. Pathological, solid aggregates (like amyloid fibrils) represent a deeper, more *thermodynamically stable* state. Healthy cells continuously invest energy (e.g., via ATP-dependent chaperones and remodelers) to maintain condensates within the functional metastable basin and prevent them from sliding into the deeper, pathological well. Factors such as pathogenic mutations (demonstrated for FUS, Tau, Huntingtin), acute cellular stress, or the chronic decline in protein quality control (PQC) associated with aging can effectively lower the kinetic barrier between these states. This “tuning” of metastability makes the LST pathway more favorable, leading to the accumulation of irreversible, toxic aggregates [40,48] (Figure 2A).

However, it is critical to challenge the binary dogma that “liquid is functional” and “solid is pathological.” Rigidity is not inherently detrimental; rather, pathology arises from the unscheduled or aberrant loss of “tunable metastability”—the formation of solids at the wrong time or place. A paradigm-shifting study by Amato et al. recently demonstrated that a precise balance of fluidity and rigidity within the centrosome is explicitly required for proper cell division, suggesting that solidified states can be essential for mechanical function [49]. This aligns with the “selective phase” model of the Nuclear Pore Complex (NPC), where FG-nucleoporins form a cohesive, hydrogel-like barrier that is functional rather than toxic [50]. Thus, the disease state is defined not merely by the material state itself (e.g., gelation), but by the loss of dynamic regulation that locks proteins into a state incompatible with their immediate physiological context. The distinct molecular grammars governing these functional fluids versus pathological solids are summarized in Table 2.

Another major TGOF mechanism involves hijacking and sequestration, where aberrant condensates become detrimental hubs. As discussed in cancer, oncogenic fusion proteins can form neomorphic condensates that sequester essential transcriptional machinery, driving aberrant gene expression [42]. In *C9orf72*-related ALS/FTD, the toxic arginine-rich dipeptide repeats form aberrant condensates that invade the nucleolus, disrupting its structure and function by sequestering key components like NPM1 [51]. Similarly, viruses construct their own condensate-based “factories” that concentrate viral replication machinery while potentially sequestering or excluding host defense factors [52].

In many severe condensatopathies, particularly neurodegenerative conditions, these distinct pathogenic mechanisms are often intertwined in a “Double-Hit” vicious cycle. TDP-43 proteinopathy serves as the archetype. Cytoplasmic TGOF (aggregation and LST) acts as a sink, leading to the depletion of functional, soluble TDP-43 from the nucleus. This nuclear depletion constitutes a critical LOF, impairing essential RNA splicing activities (e.g., for the *STMN2* gene, vital for neuronal maintenance). The consequences of this splicing failure, including potential cellular stress, may then feedback to further promote cytoplasmic aggregation, thus creating a self-perpetuating cycle of decline [37,38] (Figure 2B).

Despite the power of these conceptual models—LOF/TGOF, tunable metastability, the double-hit—a critical gap persists. We lack high-resolution, in situ structural information about these pathological assemblies within their native cellular environment. What is the precise molecular architecture of a FUS condensate undergoing LST inside a neuron? How do oncogenic fusion proteins reorganize chromatin within their aberrant transcriptional hubs? While recent advances in cryo-electron microscopy (cryo-EM) and tomography (cryo-ET) applied to patient tissues or complex cellular systems are beginning to provide unprecedented glimpses [41], these studies often capture static, potentially end-stage structures. A comprehensive understanding requires visualizing the *dynamic structural transitions* underlying pathology as they occur within the cell. Without bridging this gap—without opening the structural “black box”—our mechanistic understanding remains incomplete, hindering the development of truly structure-guided therapeutic interventions.

### 3.3. Visualizing the Invisible: Structural Insights from Cryo-ET

A major barrier to understanding condensatopathies has been the difficulty in visualizing the internal architecture of these dynamic assemblies. However, recent breakthroughs in cryo-electron microscopy (cryo-EM) and tomography (cryo-ET) are beginning to open this “black box.” For example, landmark cryo-EM studies on patient-derived tissues have overturned previous dogmas, revealing that the abundant amyloid filaments in Frontotemporal Lobar Degeneration (FTLD-FUS) are composed not of FUS, but of the distinct RNA-binding protein TAF15, establishing a new “TAF15 proteinopathy” [41].

Furthermore, in situ cryo-ET has provided glimpses into the “reversible fibers” formed by low-complexity domains (LCDs). Unlike pathogenic amyloids which form rigid “steric zippers,” functional condensates often contain labile polymers stabilized by “polar zippers” or “kinked β-sheets” as seen in FUS and hnRNPA2 assemblies [46,53]. These structures are poised at the edge of stability—the “Temperate Zone”—allowing for rapid assembly and disassembly. Visualizing these subtle structural differences between functional polymers and pathological aggregates within the cell remains the next frontier for defining the structural basis of disease.

**Table 2:** The Molecular Grammar of Material States: From Functional Fluids to Pathological Solids.

Component/System	Dominant “Grammar”/Structure	Material State	Functional vs. Pathological Context	Key Refs.
FUS/hnRNPA2	Polar Zippers/Kinked β-sheets	Dynamic Liquid/Reversible Hydrogel	Functional (Temperate Zone): Stabilized by weak polar interactions; ensures reversibility of RNP granules.	[45,46]
NPC (FG-Nups)	FG-FG hydrophobic meshwork	Selective Hydrogel (Functional Rigidity)	Functional: A cohesive, gel-like barrier required for selective nucleocytoplasmic transport; excludes inert macromolecules.	[3,4]
Centrosome	Coiled-coil scaffolds	Viscoelastic Solid (Functional Rigidity)	Functional: A precise balance of rigidity is explicitly required to withstand force during cell division.	[49]
Amyloid Fibrils	Steric Zippers	Irreversible Solid	Pathological: Dehydrated, tightly interdigitated interfaces (e.g., in Alzheimer’s) resistant to dissolution.	[44,46]
TDP-43 (Mutant)	Helix stabilization	Solid/Aggregated	Pathological: Mutations in the C-terminal IDR stabilize a transient helix, accelerating the Liquid-to-Solid transition.	[54]

## 4. The Therapeutic Conundrum: Targeting a ‘State of Matter’ in a World of ‘Active Sites’

This incomplete mechanistic picture directly feeds into the third major challenge: effective therapeutic intervention. The emergence of condensatopathies presents a fundamental conundrum for traditional pharmacology. For the better part of a century, drug discovery has operated under a paradigm centered on targeting well-defined, stable “active sites” within proteins possessing fixed three-dimensional structures—enzyme pockets, receptor binding clefts, allosteric regulatory sites. The success of this approach is undeniable, but it is predicated on a structural view of proteins that is fundamentally misaligned with the nature of condensates.

In a condensatopathy, the pathological entity is often not a mutated active site, but rather the emergent, collective “physical state” of a protein ensemble. The target might be aberrant viscosity, pathological gelation, an altered saturation concentration (Csat), the kinetics of a LST, or the promiscuous, dynamic network of interactions mediated by “stickers” within an IDR. These are properties of a *system*, not of a single, static molecular pocket. How does one design a small molecule to specifically inhibit “stickiness” or to precisely modulate “fluidity” without causing widespread off-target effects? This conceptual mismatch represents a profound obstacle.

The initial wave of “condensate-modifying drugs” (c-mods) reflects both the promise and the difficulty of this new therapeutic landscape [55]. Early strategies have logically attempted to adapt existing pharmacological principles. Some molecules function as “plasticizers,” aiming to maintain or restore condensate fluidity; lipoamide, for example, appears to dissolve stress granules through redox effects and has shown preclinical potential in ALS models [56]. Other approaches target specific “sticker” interactions deemed critical for pathological assembly. Small molecules like ET516, which covalently targets a residue involved in androgen receptor condensation, represent an attempt to disrupt the driving forces of oncogenic condensates in prostate cancer [57]. Similarly, peptides designed to interfere with the phase separation of the oncoprotein FOXM1 have shown efficacy in breast cancer models [58]. A third, more direct strategy employs targeted degradation, using tools like antisense oligonucleotides (ASOs) to reduce the synthesis of the problematic protein (e.g., ION363 targeting FUS in ALS) or proteolysis-targeting chimeras (PROTACs) to promote its destruction (e.g., targeting BRD4, a component of transcriptional condensates) [59,60].

While these pioneering efforts demonstrate that targeting condensates is feasible, they also underscore the limitations of current tools. Modulators like lipoamide may lack specificity. Interaction blockers, while potentially specific, are often developed on a laborious “case-by-case” basis and may not be generalizable. Degradation strategies, particularly ASOs and conventional PROTACs, face the critical “blunt instrument” problem: they eliminate the entire protein pool, both functional monomers and pathological assemblies. This carries a significant risk of inducing severe on-target toxicity by creating an iatrogenic Loss-of-Function state, potentially trading one pathology for another. The central challenge remains: we need therapeutic modalities specifically designed to recognize and selectively modulate the *aberrant physical state* or *assembly* of a protein, rather than simply inhibiting its canonical function or destroying it entirely. This requires a shift towards developing programmable, state-sensitive intervention platforms.

## 5. Future Directions: Towards Programmable Modulation of Cellular Phase Space

Overcoming the therapeutic conundrum necessitates moving beyond repurposing old tools and embracing strategies specifically designed for the unique challenges posed by condensatopathies. The future lies in leveraging our deepening mechanistic understanding to engineer highly specific, next-generation interventions that can precisely modulate the material state and lifecycle of targeted condensates.

One promising avenue involves refining the concept of **precise modulation of condensate homeostasis**. This goes beyond simple inhibition to include sophisticated control over assembly dynamics. The “induce-to-inhibit” strategy, exemplified by the icFSP1 molecule that triggers therapeutic FSP1 condensation to induce ferroptosis in cancer cells, showcases this potential [61]. This approach turns the pathological principle on its head, using induced phase separation as a tool for targeted inactivation. Future efforts could focus on developing molecules that fine-tune Csat, modulate interfacial tension to control droplet fusion/fission, or specifically alter viscoelastic properties to restore functional dynamics.

Another critical direction is the development of targeted degradation strategies that are sensitive to the protein’s assembly state. The goal is to eliminate the “blunt instrument” problem by selectively destroying only the pathological phase, while preserving the functional monomeric or oligomeric pool. The emergence of “state-specific” degraders, such as the TRIM21-based “TrimTACs,” represents a major conceptual advance [62]. By leveraging the natural ability of TRIM21 to recognize multimeric substrates, these degraders achieve remarkable selectivity for condensed or aggregated proteins (like activated cGAS), offering a potentially much safer therapeutic window compared to conventional PROTACs. A complementary approach involves hijacking the cell’s endogenous quality control machinery. Engineering bi-specific molecules (e.g., nanobodies) that physically link aberrant condensates or aggregates (like those formed by TDP-43) to cellular PQC hubs (such as PML bodies or autophagic receptors like p62/SQSTM1) provides a mechanism to specifically triage the pathological species for destruction [63,64].

Finally, it is worth noting that the principles governing condensate assembly and disassembly are rapidly becoming powerful tools for synthetic biology and cellular engineering. Beyond correcting pathology, we are learning to *build* with condensates. Examples include enhancing the efficacy of CAR-T cells by engineering phase-separating signaling domains [65] and constructing “Designer Membraneless Organelles” (dMLOs) capable of performing novel biochemical functions, such as orthogonal translation or metabolic channeling, within living cells [66,67]. This demonstrates a maturing ability to rationally manipulate cellular phase space for therapeutic and biotechnological purposes. An overview of these emerging therapeutic strategies and synthetic biology tools is presented in Table 3.

Beyond therapeutics, the principles of condensation are being harnessed to create novel research tools and bioengineering platforms. The “ATM-SPARK” reporter system exemplifies this: it uses a kinase-inducible phase transition to convert transient biochemical signaling (like ATM kinase activity) into robust, visible fluorescent puncta, allowing for real-time spatiotemporal tracking of signaling events. In the realm of genome engineering, tools like the “VECTOR” system utilize light-inducible condensates to apply precise physical forces to chromatin loci, enabling researchers to probe the viscoelastic properties of the genome in living cells [68]. Furthermore, “Designer Membraneless Organelles” (dMLOs) are being constructed from de novo designed proteins to act as orthogonal reaction crucibles, such as concentrating enzymes for specific metabolic pathways to enhance efficiency [69]. These innovations suggest that the future lies not just in treating condensatopathies, but in employing condensates as programmable devices for cellular control.

**Table 3:** The Next-Generation Toolkit: From Modulators to Synthetic Biology.

Category	Tool/Strategy	Mechanism of Action	Application/Potential	Key Refs.
Therapeutics: Modulators	Lipoamide	Plasticizer	Modulates redox state to maintain stress granule fluidity; restores liquidity in ALS models.	[56]
	ET516	Sticker Blocker	Covalently binds specific residues to block “sticker” interactions in Androgen Receptor condensates.	[57]
	icFSP1	Inducer (Induce-to-Inhibit)	Triggers the condensation of FSP1, sequestering it to induce ferroptosis in cancer cells.	[61]
Therapeutics: Degraders	TrimTACs	State-Specific Degrader	E3 ligase-based degrader that selectively recognizes and destroys only multimeric/condensed proteins (e.g., active cGAS).	[62]
	PQC-Engagers	Autophagy Targeting	Bi-specific nanobodies linking aggregates (e.g., TDP-43) to p62 bodies for autophagic clearance.	[64]
Synthetic Biology Tools	ATM-SPARK	Biosensor	Kinase activity triggers a phase transition, converting a transient signal into a visible fluorescent punctum for real-time tracking.	[70]
	VECTOR	Genomic Force Probe	Uses light-inducible condensates to exert physical force on chromatin, probing genomic viscoelasticity.	[69]
	dMLOs	Designer Organelles	De novo designed condensates that compartmentalize enzymes (e.g., for orthogonal translation) to enhance reaction efficiency.	[66,67]

## 6. Conclusions and Perspective: Embracing a New Cycle of Discovery and Intervention

The discovery of LLPS and biomolecular condensates has irrevocably changed our understanding of cellular organization, revealing a dynamic layer of control governed by the principles of soft matter physics. This “material science” perspective has, in turn, illuminated the origins of a vast spectrum of human diseases—the Condensatopathies—arising from the dysregulation of this delicate cellular phase space.

However, the path from this conceptual revolution to tangible clinical impact requires overcoming significant hurdles. As outlined in this review, critical gaps remain in our ability to comprehensively discover the full range of proteins and pathways involved, to fully analyze the precise structural and mechanistic underpinnings of pathological transitions in situ, and to effectively intervene using tools specifically designed to target the unique physical nature of condensates.

Addressing these challenges demands a new, integrated research paradigm—a self-reinforcing “Discover–Analyze–Intervene–Rediscover” cycle. Progress will depend on the synergistic application of cutting-edge technologies. Artificial intelligence and high-throughput multi-omics will be essential for systematic, unbiased *Discovery*, allowing us to map the true breadth of the disease condensatome. In situ structural biology, particularly cryo-electron tomography, will be indispensable for *Analysis*, providing the high-resolution “ground truth” needed to unravel complex pathogenic mechanisms. And synthetic and chemical biology will be crucial for developing the next generation of *Interventions*, moving beyond conventional pharmacology towards programmable, state-sensitive modulators.

The “programmable dissolvers” concept, based on reverse-engineering inhibitory IDRs, exemplifies the power of this integrated approach. Such a tool is not merely a potential therapeutic; it is also a uniquely powerful research probe. Its ability to precisely dismantle specific condensates in living systems offers an unprecedented method for establishing causal links between structure, function, and pathology, thereby directly addressing the mechanistic gap. Furthermore, observing the cellular response to such targeted perturbations will inevitably reveal new regulatory networks and compensatory pathways, fueling further rounds of discovery.

By embracing this iterative cycle—using advanced tools for discovery, deep structural analysis to inform intervention design, and then deploying those interventions as probes to drive further discovery—the field of biomolecular condensates is poised to transition from observation to rational engineering. This journey promises not only to deepen our fundamental understanding of life’s organizational principles but also to establish the modulation of cellular phase space as a central and transformative pillar of 21st-century precision medicine.

## Data Availability

No new data were created or analyzed in this study. Data sharing is not applicable to this article.

## Abbreviations

ALS	Amyotrophic Lateral Sclerosis
AML	Acute Myeloid Leukemia
ASO	Antisense Oligonucleotide
CAR	Chimeric Antigen Receptor
c-mod	Condensate-modifying drug
cryo-ET	Cryo-electron Tomography
Csat	Saturation Concentration
dMLO	Designer Membraneless Organelle
FTD	Frontotemporal Dementia
IDR	Intrinsically Disordered Region
iIDR	Inhibitory Intrinsically Disordered Region
LCD	Low-complexity Domain
LLPS	Liquid–liquid Phase Separation
LOF	Loss-of-Function
LST	Liquid-to-Solid Transition
pIDR	Promoting Intrinsically Disordered Region
PQC	Protein Quality Control
PROTAC	Proteolysis-targeting Chimera
SMA	Spinal Muscular Atrophy
TGOF	Toxic Gain-of-Function
